# Calcium and magnesium content of the uterine fluid and blood serum during the estrous cycle and pre-pubertal phase in water buffaloes 

**Published:** 2014

**Authors:** Sayed Mortaza Alavi Shoushtari, Siamak Asri Rezaie, Amir Khaki, Abulfazle Belbasi, Hamid Tahmasebian

**Affiliations:** *Department of Clinical Sciences, Faculty of Veterinary Medicine, Urmia University, Urmia, Iran.*

**Keywords:** Buffalo, Calcium, Estrous cycle, Magnesium, Uterine fluid

## Abstract

To investigate uterine fluid and serum calcium (Ca) and Magnesium (Mg) variations during the estrus cycle in water buffaloes, 71 genital tracts and blood samples were collected from the abattoir in Urmia. The phase of the estrous cycle was determined by examining ovarian structures. 18 animals were pro-estrous, 15 estrous, 16 met-estrous and 22 diestrous. The uterine fluid was collected by gentle scraping of the uterine mucosa with a curette. Blood serum and uterine fluid samples of 71 pre-pubertal buffalo calves were also collected and treated in similar manners. The mean ± SEM total serum and uterine fluid Ca in cyclic buffaloes were 8.68 ± 0.28 mg dL^-1^ and 8.10 ± 0.2 mg dL^-1^ *vs.* 6.76 ± 0.65 mg dL^-1^ and 7.90 ± 0.15 mg dL^-1^ in pre-pubertal calves, respectively. Blood serum Mg was not different in cyclic and pre-pubertal animals but the uterine fluid Mg in cyclic cows was higher than those in pre-pubertal calves. Serum Ca in pro-estrus and estrus were higher than those in other stages and also higher than those in the uterine fluid. The lowest Mg content of serum was recorded in diestrus, while in the uterine fluid it was observed in estrus. In all stages of estrous cycle except for estrus the uterine fluid Mg content was significantly higher than those of the serum. These results suggested that during the estrous cycle in the buffalo cows, Ca was passively secreted in uterine lumen and mostly dependent on blood serum Ca concentrations but Mg was secreted independently. The values (except for serum total Mg) also increased after puberty.

## Introduction

There are two intracellular and extracellular calcium (Ca) sources in the body and the skeleton is a major reservoir for providing Ca for both the extra- and intra-cellular pools. The role of Ca in cell functions, including spermatozoa has been reported.^1^^-^^7^

Magnesium (Mg), the second most prevalent intra-cellular cation, is involved in the metabolic activity of the cell.^1^ Intracellular Mg is involved in the activity of hormone receptor complex in the cell membrane. ^4^

Both Ca and Mg ions move through epithelial cells of the uterus into the lumen of the reproductive tract causing a concentration gradient which causes an osmotic gradient providing the driving force to transport water by osmosis out of the epithelial cells into the uterine lumen. The ionic composition of uterine fluid is apparently derived from a combination of ions from blood and ions secreted from uterine epithelium.^8^ Leese points out that ion concentration and their movements are essential for the regulation of enzyme activity and of the pH of the uterine fluid. ^9^

Endometrium has different cell structure which secrete a variety of different materials into the uterine lumen.^10^ Cyclicity has an important role in protection of reproductive tract against infections. This protection in uterus is created by several mechanisms, such as increased uterine motility^11^ and secretion of immunoglobulins in the uterine lumen in estrus,^12^ in which the defense mechanisms are enhanced by plasma estradiol concentration and help in clearance of infectious agents.^13^^-^^17^ Oviduct and uterine secretions are necessary for spermatozoa capacitation and oocyte maturation and for zygote survival.^18^^-^^21^ The composition of these secretions is an important guide in preparing medium for *in vitro* fertilization and embryo culture.

A comparison of serum Ca and Mg levels in normal cyclic, repeat breeder and anestrous buffalo cows,^22^ in follicular fluid of small and large follicles,^23^^,^^24^ and in oviduct fluid of buffalo cows at different stages of estrous cycle has been reported.^25^ However, despite the importance of Ca and Mg ions in uterine fluid formation^26^ and in gamete motility,^27^^-^^29^ zygote and early embryo development,^30^ there is little published information on the Ca and Mg concentrations in the uterine fluid during the estrous cycle in buffalo cows particularly at their pre-pubertal phase. 

This work was carried out to investigate Ca and Mg concentration variations in the blood serum and uterine fluid during different phases of the estrous cycle and at pre-pubertal phase in buffaloes searching for any possible inter-relationship. 

## Materials and Methods

Genital tract and blood sample of 300 female slaughter buffaloes with unknown history of reproduction and state of nutrition were collected from Urmia abattoir (37˚ 33΄ N, 45˚ 4΄ E) from November 2011 to June 2012. Body condition scores (Score 2.5 to 4) and ages of the animals (2 to 4 years) had been recorded before sample collection. Samples of animals with body condition scores (BCS) of less than 2.5 and more than 4 (on the scale of: 1 = emaciated to 5 = obese) were not collected. Samples were quickly transferred to the lab in a cold box. On an initial examination, all the abnormal and pregnant samples were discarded. Genital tracts were examined to determine the stage of their cycles and pre-mature states by examining the structures on their ovaries described by Noakes.^31^ Out of normal cyclic genital tracts, 18 pro-estrous, 15 estrous, 16 met-estrous and 22 diestrous samples, which had clear (non-hemolyzed) serum samples, were finally used. Due to some problems, serum progesterone assay was not carried out. Seventy one pre-pubertal genital tracts of heifers with BCS 1.5 to 3 and 1 to 1.5 years old were also selected. Blood samples were collected by jugular vein puncture in plain glass tubes before slaughter and allowed to clot. Serum samples were harvested by centrifuging the clotted blood at 3000 rpm for 10 minutes and stored in Eppendorf micro-tubes at –20 ˚C until analysis. Hemolyzed blood samples and their related genital tracts were discarded.

Uterine fluid samples were collected by gentle scraping of the mucosa by a curette after incision of the uterine horns (both ipsi- and contra-lateral side to corpus luteum, if present) and stored in Eppendorf micro-tubes at –20 ˚C until examination. Uterine fluid and blood serum Ca and Mg contents of the samples were determined by a spectrophotometry method using commercial Ca and Mg assay kits (ELITech Group, Puteaux, France) after thawing the samples. 

Data was analyzed using PAWS software (Version 18; SPSS Inc., Chicago, USA). Data was analyzed by one way ANOVA. Statistic means and standard error of mean were calculated for each group. Serum and uterine fluid samples in the groups were compared by paired Student’s *t*-test and the limit for statistical significance was set at *p *≤ 0.05. Pearson coefficient test was used to determine correlations between serum and uterine values. 

## Results

The mean (± SEM) total Ca value obtained from all the 71 serum and uterine fluid of cyclic samples were 8.68 ± 0.28 and 8.10 ± 0.2 mg dL^-1^, respectively, both were significantly higher (*p* < 0.01) than those obtained for pre-pubertal samples (6.76 ± 0.65 and 7.90 ± 0.15 mg dL^-1^, respectively), (Table 1). Total serum Ca (r^2^ = 0.699, *p* = 0.000) and Mg (r^2 ^= – 0.443, *p* = 0.000) were also correlated with the uterine fluid total Ca and Mg values. The mean serum Ca concentration was 11.09 ± 0.52 mg dL^-1 ^in pro-estrus; and 8.57 ± 0.74 mg dL^-1^, 6.99 ± 0.22 mg dL^-1 ^and 8.02 ± 0.24 mg dL^-1 ^in estrus, met-estrus, and diestrus, respectively. Serum Ca concentration in pro-estrus was significantly (*p* < 0.05) higher than those in other stages. The mean Ca concentration in uterine fluid samples was 9.45 ± 0.39 mg dL^-1^ in pro-estrus. It was recorded 6.63 ± 0.30 mg dL^-1 ^in estrus, 7.21 ± 0.23 mg dL^-1^ in met-estrus, and 8.63 ± 0.33 mg dL^-1 ^in diestrus. The mean uterine fluid Ca concentration in pro-estrus and diestrus were different (*p* < 0.05) from those of the other phases (Table 2). Significant correlations were observed within serum Ca values in pro-estrus, estrus and met-estrus samples but they were not correlated with those in the uterine fluid samples. 

**Table 1:** Serum and uterine fluid total Ca and Mg concentrations in cyclic and pre-pubertal samples. Data are presented as mean ± SEM

**Parameters**	**No.**	**Cyclic**	**Pre-pubertal**	***p*** **-value**
***Serum***				
**Ca ** **(mg** ** dL** ^-1^ **)**	71	8.68 ± 0.28*	6.76 ± 0.65	0.002
**Mg ** **(mg** ** dL** ^-1^ **)**	71	4.43 ± 0.19	4.27 ± 0.21	0.557
***Uterine fluid***				
**Ca ** **(mg** ** dL** ^-1^ **)**	71	8.10 ± 0.2*	7.90 ± 0.15	0.008
**Mg ** **(mg** ** dL** ^-1^ **)**	71	9.47 ± 0.26*	7.01 ± 0.24	0.000

* indicates difference in the same row at *p* < 0.01 level.

The mean serum and the uterine fluid total Mg content of cyclic samples were 4.43 ± 0.19 mg dL^-1 ^and 9.47 ± 0.26 mg dL^-1^. These values in pre-pubertal samples were recorded 4.27 ± 0.21 mg dL^-1 ^and 7.01 ± 0.24 mg dL^-1^, respectively, and were significantly different (*p* < 0.01) only from uterine fluid values in cyclic samples (Table 1). The mean serum Mg concentration in pro-estrus was 5.16 ± 0.31 mg dL^-1^. It was 5.71 ± 0.48 mg dL^-1^, 4.00 ± 0.32 mg dL^-1 ^and 3.28 ± 0.15 mg dL^-1 ^in estrus, met-estrus and diestrus, respectively (Table 3). The mean Mg concentration in the uterine fluid samples was recorded 8.80 ± 0.18mg dL^-1 ^in pro-estrus; it was 6.21 ± 0.38 mg dL^-1^, 11.06 ± 0.26 mg dL^-1 ^and 11.07 ± 0.18 mg dL^-1 ^in estrus, met-estrus and diestrus, respectively. Serum Mg contents in all the stages of the cycle except in estrus were significantly (*p* < 0.001) lower than those of the uterine fluids (Table 3). No significant correlation was observed between serum and uterine fluid Mg values in different stages of the cycle. 

Serum Ca concentration in pro-estrus (11.09 ± 0.52 mg dL^-1^) was significantly higher than the values in all other stages. Calcium content of the uterine fluid in pro-estrus (9.45 ± 0.39 mg dL^-1^) and diestrus (8.63 ± 0.33 mg dL^-1^) were higher than Ca values in estrus and met-estrus samples. It was noticed that in pro-estrus and estrus phases of the cycle Ca concentrations in the serum were higher (*p* < 0.05) than those in the uterine fluid, but in met-estrus, and diestrus Ca content of the uterine fluid was higher (though not statistically significant), (Table 2). 

The highest serum Mg value (5.71 ± 0.48 mg dL^-1^) observed in estrus and the lowest (3.28 ± 0.15 mg dL^-1^) in diestrus, were significantly different. Also, the highest Mg concentration in the uterine fluid samples observed in met-estrus and diestrus (11.06 ± 0.26 and 11.07 ± 0.18 mg dL^-1^) were significantly different from the lowest value in estrus (6.21 ± 0.38 mg dL^-1^). The mean Mg concentrations of serum or uterine fluid samples in all the stages but estrus were significantly different (*p* < 0.001), and the values in uterine fluid samples were higher than those of the serum samples (Table 3).

**Table 2:** Serum and uterine fluid Ca concentrations in different phases of estrus cycle of the buffalo cow. Data are presented as mean ± SEM

**Phases of the cycle**	**No.**	**Serum** **(mg dL** ^-1^ **)**	**Uterine fluid** **(mg dL** ^-1^ **)**
**Pro-estrus**	18	11.09 ± 0.52^a^*	9.45 ± 0.39^a^
**Estrus**	15	8.57 ± 0.74^b^*	6.63 ± 0.30^b^
**Met-estrus**	16	6.99 ± 0.22^b^	7.21 ± 0.23^b^
**Diestrus**	22	8.02 ± 0.24^b^	8.63 ± 0.33^a^

* indicates difference in the same row at *p* < 0.05 level.

a,b Different superscripts indicate significant difference in the same column (*p* < 0.05).

**Table 3:** Serum and uterine fluid Mg concentrations in different phases of estrus cycle of the buffalo cow. Data are presented as mean ± SEM

**Phases of the cycle**	**No.**	**Serum** **(mg dL** ^-1^ **)**	**Uterine fluid** **(mg dL** ^-1^ **)**
**Pro-estrus**	18	5.16 ± 0.31^ab^*	8.80 ± 0.18^a^
**Estrus**	15	5.71 ±0.48^a^	6.21 ± 0.38^b^
**Met-estrus**	16	4.00 ± 0.32^b^*	11.06 ± 0.26^c^
**Diestrus**	22	3.28 ± 0.15^c^*	11.07 ± 0.18^c^

* indicates difference in the same row at *p* < 0.001.

a,b,c Different superscripts indicate significant difference in the same column (*p* < 0.05).

## Discussion

Serum and uterine fluid Ca and Mg contents in buffaloes vary during the estrous cycle but somehow different from that previously reported in the bovine.^6^^,^^32^^-^^34^ Different methods of collecting uterine fluid samples have been used by other workers which had some problems such as condensation of the flushing or imposing a surgical operation on the animal. The method we used in this study had none of these problems and the secretions were directly underwent analyses.

The mean Ca value obtained in this study for total serum samples (8.68 ± 0.28 mg dL^-1^) was nearly the same as that (9.30 ± 0.29 mg dL^-1^) reported by Chaurasia *et al*. for blood serum Ca content of normal cyclic buffalo cows, and the values of 8.02 ± 0.30 mg dL^-1 ^reported by Arshad *et al*. and 9.52 ± 0.17 mg dL^-1^ reported by Tabatabaei and Mamoei for buffalo plasma Ca content.^22^^-^^24^ Lower serum and uterine fluid total Ca and Mg content in pre-pubertal buffaloes observed in this study suggest that hormonal changes that occur at puberty may have stimulatory effects to increase concentration of these ions in both the blood serum and the uterine fluid.

The observed serum total Ca value in this study was lower than the mean normal value of 10.20 ± 0.28 mg per 100 mL reported by Hoffman and 9.70 to 12.40 mg dL^-1 ^reported by Radostits *et al*. for bovine blood plasma calcium content.^35^^,^^36^ These were in agreement with the values of 8 to 10.5 mg dL^-1 ^in the bovine blood serum reported by Blood and Radostits and 9.58 ± 0.14 mg dL^-1 ^reported by Das *et al*. in crossbred cattle.^37^^,^^38^ It has been pointed out that there may be variations in plasma Ca concentrations as a result of reproductive state, age or plain of nutrition of the animals.^35^^-^^37^ Effects of age and state of nutrition on Ca level in this study were minimized by collecting samples of animals in certain ranges of age and body condition.

The highest serum Ca content was observed in pro-estrus (11.09 ± 0.52 mg dL^-1^) and a low level (8.02 ± 0.24 mg dL^-1^) in diestrus, which were significantly different (Table 2). This was contrary to the report of Jordan *et al*. that bovine blood plasma Ca content increased with elevations in plasma progesterone concentration,^39^ and in agreement with the report of Hugentobler *et al*. who found no significant difference in bovine serum Ca at days 2 (met-estrus) and 14 (diestrus) of the cycle.^8^ A significant difference of the serum Ca concentration in pro-estrus with those of the other stages observed here could be consequences of the hormonal changes that occur in this stage of the estrous cycle.

The highest uterine fluid Ca content (9.45 ± 0.39 mg dL^-1^) was observed in pro-estrus and the least (6.63 ± 0.30 mg dL^-1^) in estrus, in which the difference was significant (*p* < 0.05). It was also significantly different from that observed in met-estrus that was also contrary to the report of Jordan *et al*. ^39^ The least uterine fluid Ca and Mg content observed in estrus in buffaloes might be caused by their dilution in the uterine secretions which occur in this stage as a result of increased uterine blood flow induced by estradiol or could be due to the negative effect of this hormone. It was noticed that uterine fluid Ca content in pro-estrus and estrus was lower than that of the serum and followed its changes, which could be explained by its passive secretion in the uterine lumen from endometrial capillaries as suggested by Aitken in roe deer.^40^ The situation, however, was reversed in met- and diestrus that the uterine fluid Ca was higher (though not significant).

Serum total Mg contents observed in this study (4.43 ± 0.19 mg dL^-1 ^in cyclic cows and 4.27 ± 0.21 mg dL^-1 ^in pre-pubertal calves) were higher than those observed in cattle by Hoffman who reported that the mean value of bovine blood plasma Mg is 2.89 ± 0.25 mg per 100 mL,^35^ Blood and Radostits who reported 1.2 to 3.5 mg dL^-1 ^in the bovine blood serum^37^ and Radostits *et al*. who reported the mean value of bovine serum Mg 1.80 to 2.30 mg dL^-1^.^36^

The highest value for serum Mg concentration was observed in estrus (5.71 ± 0.48 mg dL^-1^), which was not significantly different from those in other stages of the cycle but diestrus (3.28 ± 0.15 mg dL^-1^), which was the lowest value. This is in agreement with the report of Hugentobler *et al*. who found no significant difference in bovine serum Mg during the stages of the cycle,^8^ but contrary to their finding of the highest serum Mg value in diestrus, and to the report of Jordan *et al*. that blood plasma Mg content increased with elevations in plasma progesterone concentration.^39^ Our result of the highest uterine fluid Mg content in met- and diestrus (11.06 ± 0.28 mg dL^-1^ and 11.70 ± 0.18 mg dL^-1^) and a low Mg content in estrus is in agreement with the reports of Hugentobler *et al*. who found higher uterine fluid Mg content in diestrus in the bovine^8^ and with the report of Jordan *et al*. who found that uterine fluid Mg content increased with an increase in serum progesterone concentrations.^39^ In this study the higher Mg content of uterine fluids in all the stages of the cycle than those of the serum was in agreement with the report of Hugentobler *et al*.^8^

These results indicated that in buffaloes, like bovine, calcium secretion in uterine fluid followed the changes which occurred in serum calcium content during the phases of the estrous cycle and it was usually lower than that of the serum values. However, magnesium levels in the uterine fluid were higher than those in the serum and were not changed with the variations in the serum values at different stages of the cycle, suggesting that its secretion in the uterine lumen was possibly an active one. 
